# Correction: Synthesis and optical properties of lead-free cesium germanium halide perovskite quantum rods

**DOI:** 10.1039/c9ra90076d

**Published:** 2019-10-21

**Authors:** Lin-Jer Chen

**Affiliations:** Department of Photonics, National Cheng Kung University Tainan 70101 Taiwan linjerchen@gmail.com

## Abstract

Correction for ‘Synthesis and optical properties of lead-free cesium germanium halide perovskite quantum rods’ by Lin-Jer Chen, *RSC Adv.*, 2018, **8**, 18396–18399.

The affiliation in the original article was incorrect. The correct affiliation is listed herein.

The author apologises that there are portions of text overlap between this *RSC Advances* article and their previous related paper published in *Journal of Physical Chemical Letters*, cited as ref. 21 in the original article.

In addition, there is an error in the ‘Characterization and measurements’ section of the ESI. The first sentence should be changed from “The as-prepared samples were characterized by X-ray powder diffraction (XRD), and transmission electron microscopy (TEM)” to “The as-prepared samples were characterized by X-ray photoelectron spectroscopy (XPS), and transmission electron microscopy (TEM)”. There was no XRD data reported, therefore the ESI has been updated online to reflect this change.

The Royal Society of Chemistry apologises for these errors and any consequent inconvenience to authors and readers.

## Supplementary Material

